# Classification and genetic counselling for a novel splicing mutation of the *MLH1* intron associated with Lynch syndrome in colorectal cancer

**DOI:** 10.1093/gastro/goab030

**Published:** 2021-09-06

**Authors:** Ling-Ling Wang, Shuang-Mei Zou, Lin Dong, Ming Yang, Dan Qi, Zhao Lu, Jia-Nan Chen, Shi-Wen Mei, Zhi-Xun Zhao, Xu Guan, Zheng Jiang, Qian Liu, Zheng Liu, Xi-Shan Wang

**Affiliations:** 1 Department of Colorectal Surgery, National Cancer Center/National Clinical Research Center for Cancer/Cancer Hospital, Chinese Academy of Medical Sciences and Peking Union Medical College, Beijing, P. R. China; 2 Department of Pathology, National Cancer Center/National Clinical Research Center for Cancer/Cancer Hospital, Chinese Academy of Medical Sciences and Peking Union Medical College, Beijing, P. R. China

**Keywords:** Lynch syndrome, MLH1, genetic testing, colorectal cancer, genetic counselling

## Abstract

**Background:**

Lynch-syndrome-associated cancer is caused by germline pathogenic mutations in mismatch repair genes. The major challenge to Lynch-syndrome screening is the interpretation of variants found by diagnostic testing. This study aimed to classify the *MLH1* c.1989 + 5G>A mutation, which was previously reported as a variant of uncertain significance, to describe its clinical phenotypes and characteristics, to enable detailed genetic counselling.

**Methods:**

We reviewed the database of patients with Lynch-syndrome gene detection in our hospital. A novel variant of *MLH1* c.1989 + 5G>A identified by next-generation sequencing was further investigated in this study. Immunohistochemical staining was carried out to assess the expression of MLH1 and PMS2 protein in tumour tissue. *In silico* analysis by Alamut software was used to predict the *MLH1* c.1989 + 5G>A variant function. Reverse transcription-polymerase chain reaction and sequencing of RNA from whole blood were used to analyse the functional significance of this mutation.

**Results:**

Among affected family members in the suspected Lynch-syndrome pedigree, the patient suffered from late-stage colorectal cancer but had a good prognosis. We found the *MLH1* c.1989 + 5G>A variant, which led to aberrant splicing and loss of MLH1 and PMS2 protein in the nuclei of tumour cells. An aberrant transcript was detectable and skipping of *MLH1* exon 17 in carriers of *MLH1* c.1989 + 5G>A was confirmed.

**Conclusions:**

*MLH1* c.1989 + 5G>A was detected in a cancer family pedigree and identified as a pathological variant in patients with Lynch syndrome. The mutation spectrum of Lynch syndrome was enriched through enhanced genetic testing and close surveillance might help future patients who are suspected of having Lynch syndrome to obtain a definitive early diagnosis.

## Introduction

Colorectal cancer (CRC) is the third most common cancer in the world, with the third highest incidence and the second highest mortality rate [1]. It is estimated that CRC accounted for 9.39% of deaths from all cancers in 2020, and >1.93 million new cases and 0.94 million deaths have been reported worldwide [1]. Thirty per cent of patients have a family history of CRC and this is related to the interaction between heredity and the environment [2]. Lynch syndrome (LS) is the main cause of hereditary CRC and accounts for ∼3% of all new diagnoses of CRC [3]. The 10-year crude survival rate after CRC diagnosis is 91% and the crude survival rate for any tumour secondary to LS is 82% [4]. LS is an autosomal dominant genetic disorder caused by germline mutations in mismatch repair (MMR) genes, including *MLH1*, *MSH2*, *MSH6*, *PMS2*, and *EPCAM* [5]. LS is associated with an increased risk of cancer, especially CRC, ranging from 40% to 80% [6]. In a previous report, carriers with the *MLH1* pathogenic mutation had a 46% cumulative incidence of developing CRC by 75 years of age [7]. Additionally, due to deficient mismatch repair (dMMR), LS patients also have a high risk for a second primary tumour [8]. Therefore, the timely and definitive diagnosis of LS facilitates the management and intervention of patients and family members to reduce the incidence of cancer. In recent years, the use of DNA genetic testing to identify germline mutations and diagnose hereditary cancer has been widely accepted. Genetic testing is also the most effective method for diagnosing LS [9]; through testing, an increasing number of mutations are being recognized [10]. Classifications of mutations include pathogenic, likely pathogenic, variants of uncertain significance (VUS), likely benign, and benign [10]. It has been reported that 5%–40% of variants are classified as VUS [9]. VUS has gradually become a clinical challenge due to the undetermined significance of these variants in terms of cancer risk.

Therefore, identifying and classifying variants with clinical significance among suspected LS patients are important to efficiently screen and manage at-risk carriers with pathogenic variants. In this study, we report a variant of *MLH1*, namely c.1989 + 5G>A, in a suspected LS family pedigree, which was previously described as a VUS in the database and has no confirmation of pathogenicity. As is usual, variants located in the first two base pairs (±1/2 bp) of introns, which were highly likely to disrupt splicing, were taken into consideration for pathogenicity; however, the c.1989 + 5G>A mutation occurs in the fifth position of the intron, which few genetic analysts would pay attention to.

Here, we examine the significance of this variant using molecular analysis, make a definitive diagnosis, and provide information for meaningful genetic counselling and follow-up for all carriers of these mutated families.

## Materials and methods

### Patients

We reviewed the patient database for LS-gene detection at the centre of Molecular Pathology, Cancer Hospital, Chinese Academy of Medical Sciences between January 2016 and October 2020. Three patients carried this mutation, two of whom were from the same family and one of whom was from another family. Because the index patient from the second family had undergone neoadjuvant immunotherapy and achieved pathological complete response (pCR), we performed the analysis only on the first family but provided clinical information for both families. The proband in the first family was pathologically diagnosed with rectal cancer with loss of MLH1 and PMS2 found by immunostaining in our hospital. The patient’s mother, uncle, and aunt also developed intestinal tumours. Immunohistochemistry (IHC) of the uncle’s tumour tissue showed the same MMR protein deficiency as the proband. All procedures in this study involving human participants conformed to the ethical standards of the Ethics Committee of the Cancer Hospital of the Chinese Academy of Medical Sciences and the 2014 Declaration of Helsinki. Informed consent was obtained from all individual participants included in the study. The study was approved by the Ethics Committee of NCC/CICAMS (NCC1790).

### IHC of MMR proteins

IHC analyses of MMR proteins, including MLH1, PMS2, MSH2, MSH6, and BRAF V600E, were routinely performed in CRC patients. Monoclonal antibodies against MLH1 (clone ES05), PMS2 (clone EPR3947), MSH2 (clone FE11), MSH6 (clone EP49) (Beijing Zhongshan Golden Bridge Biotechnology, China), and BRAF V600E (VE1) (Ventana Medical Systems, AZ, USA) were used. Immunohistochemical results were assessed by two pathologists. The absence of nuclear staining in tumour cells or very faint nuclear staining in focal tumour cells was defined as loss of protein expression (i.e. dMMR).

### DNA and RNA extraction

Formalin-fixed and paraffin-embedded tumour tissues and saliva were collected for DNA extraction using a TGuide Genomic DNA One-Step Kit using a TGuide automated Nucleic Acid Preparation Instrument (TIANGEN BIOTECH, Beijing, China) according to the manufacturer’s instructions. Total RNA was extracted from the peripheral blood of patients using TRIzol (Agilent Technologies, USA). The quality and concentration of DNA and RNA were determined using a NanoDrop 2000 fluorescence spectrophotometer (NanoDrop; Thermo Science, Waltham, MA, USA).

### Microsatellite instability test

The fluorescent polymerase chain reaction was performed with a series of primers against single nucleotide repeats (NR-21, NR-24, NR-27, BAT-25, BAT-26, and MONO-27) (MSI-Reader MSI Analysis System, MICROREAD, Beijing, China). If there were two or more shifts in these six microsatellite markers, it was defined as microsatellite instability-high (MSI-H).

### Next-generation sequencing

For MMR gene germline analysis, targeted DNA sequencing was performed. DNA was profiled using a capture-based targeted sequencing panel covering 53 genes, including MMR genes (Burning Rock Biotech, Guangzhou, China). The established indexed libraries were sequenced on a MiSeq (Illumina, San Diego, CA, USA) with paired-end reads at an average depth of 500×. Sequencing data were mapped to the human genome (hg19) using BWA aligner 0.7.10. Local alignment optimization and variant calling were performed using GATK v3.2–2. Identified single-nucleotide variants (SNVs) and indels were annotated using the dbNSFP (v30a), COSMIC (v69), and dbSNP (snp138) databases. The identified *MLH1* variant by next-generation sequencing (NGS) test was also confirmed by Sanger sequencing using specific primers in the proband and family pedigree.

### Model prediction in identifying the risk of having LS

PREMM5 uses patient demographics, personal and family history of cancer, and the types of cancer with ages of diagnosis to predict the chance of having an LS mutation. A probability score of >2.5% was considered to be supportive of a clinical referral of the individual for genetic testing. The PREMM5 algorithm is available online at http://premm.dfci.harvard.edu/ and has been made available for use at no cost by the Dana-Farber Cancer Institute.

### *In silico* prediction of functional impact

*MLH1* variants were analysed using several bioinformatic tools (Align GVGD, SIFT, PolyPhen2, MutationTaster) to evaluate the impact on protein function. Splice prediction of the variant was evaluated bioinformatically using Alamut software that includes SpliceSiteFinder (SSF), MaxEntScan, NNSplice, and GeneSplicer (http://www.interactive-biosoftware.com/alamut.html).

### mRNA splicing analysis assay

Reverse transcription-polymerase chain reaction (RT-PCR) was performed using a PrimeScript II 1st Strand cDNA Synthesis Kit (Takara, Japan). Amplification of the *MLH1* coding region containing the splicing mutation was performed with the following specific primers: exon 15-F, 5′-TGAAGAACTGTTCTACCAGATAC-3′; exon 18-R, 5′-AAACATTCCTTTTCTTCGTCCCA-3′. The PCR product was separated by an electrophoresis gel and purified (TIANgel Midi Purification Kit; TIANGEN BIOTECH, Beijing, China). The expected products were subjected to Sanger sequencing on an Applied Biosystems 3500 Genetic Analyser.

## Results

### Characteristics of the family

**Figure 1.**
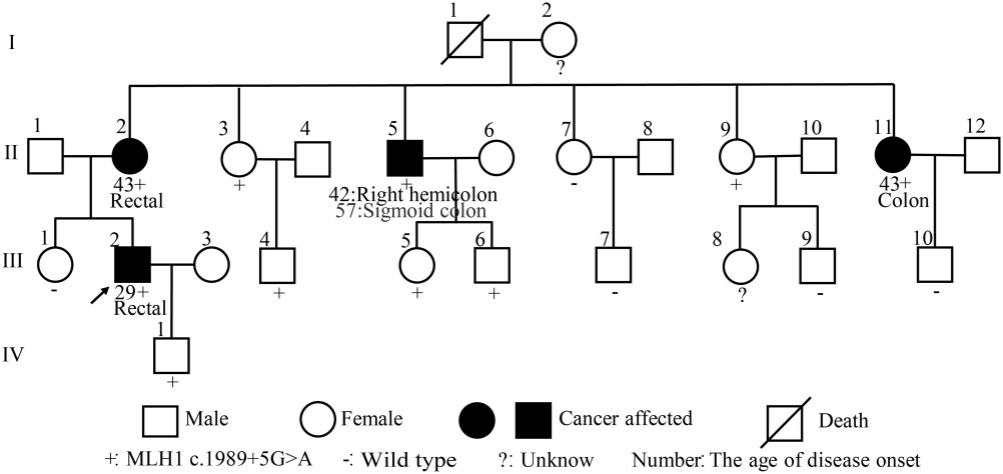
Pedigree of the proband

The proband (III : 2) from the first family was diagnosed with rectal cancer with TNM stage III at the age of 29 years. He received surgical resection and post-operative adjuvant chemotherapy and radiotherapy. His family history fulfilled the Amsterdam II and revised Bethesda clinical diagnostic criteria, as shown in the family pedigree (Figure 1). Four members (III : 2, II : 5, II : 11, III : 2) of the family suffered from CRC but had no other tumour history. The average age of disease onset in this family was 39 years. All four patients in this family underwent curative resection and no metastasis was detected in the critical follow-up window of 60 months. The aunt of the proband from the second family had an intestinal tumour in her 40s and the proband’s grandmother had a brain tumour in her 50s. This colon-cancer onset age of the proband was 24 years; the tumour was staged as T4NxM1 (stage IV) and the patient achieved a significant benefit from eight cycles of oxaliplatin combined with PD1 inhibitor therapy. The patient’s post-operative pathology stage was ypT0N0. The primary and metastatic lesions had disappeared.

### Clinical prediction algorithm for LS

PREMM5 is a clinical prediction algorithm that comprehensively assesses LS probability. Based on the information of the proband’s personal and family history, the overall predicted probability of the proband carrying a germline mutation in the *MLH1*, *MSH2*, *MSH6*, *PMS2*, or *EPCAM* genes was ≥50% in the PREMM5 model. Considering that the overall predicted probability was ≥2.5%, a recommendation for referral of the proband for genetic testing was supported.

### Analysis of MMR proteins and microsatellite instability status

**Figure 2.**
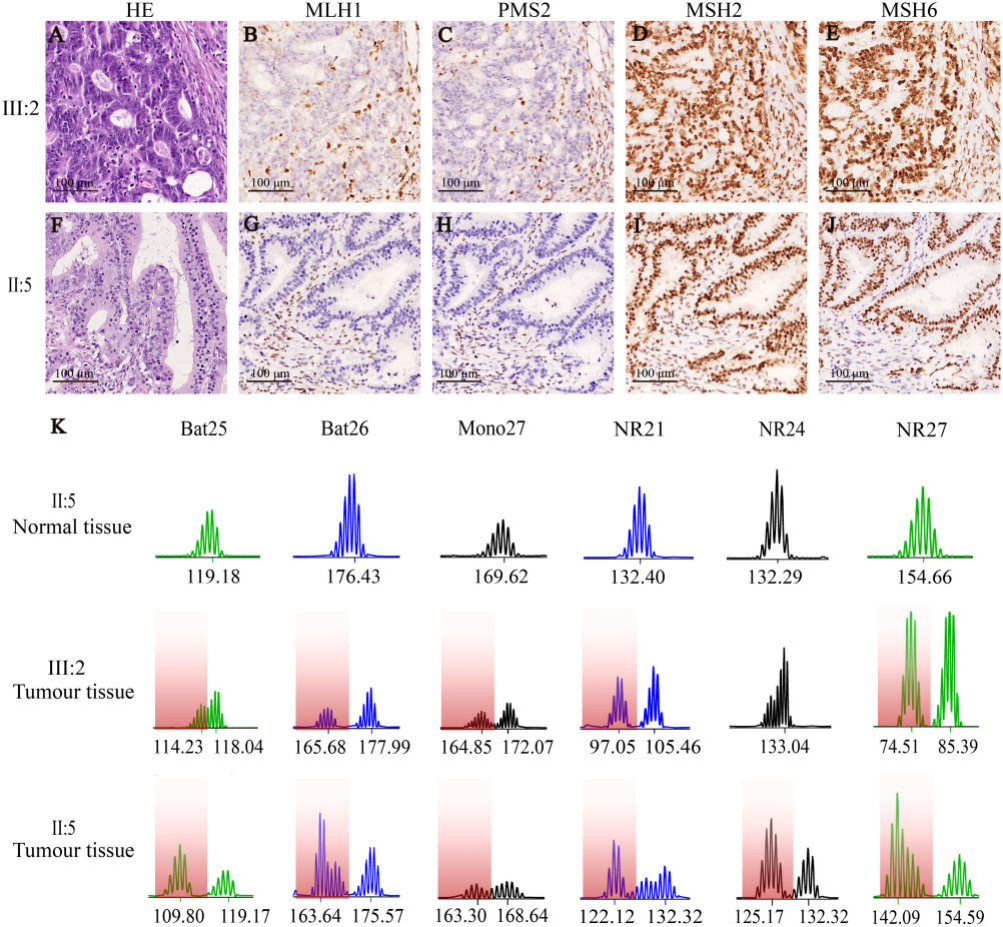
Haematoxylin and eosin (HE), immunohistochemistry (IHC), and microsatellite instability (MSI) in III : 2 and II : 5. (A)–(J) original magnification, ×200. (A) and (F) HE staining in III : 2 and II : 5 tumour tissues. (B)–(E) and (G–J) IHC in III : 2 and II:5. From left to right, IHC represents MLH1, PMS2, MSH2, and MSH6. (K) Amplification profile from patients III : 2 and II : 5 showing MSI with variant alleles that are beyond the quasimonomorphic range in tumour tissues (the coloured areas). Due to the microsatellite of normal tissue being all stable in patients III : 2 and II : 5, we only show the MSI of normal tissue in one patient.

IHC staining of the rectal tumour tissue of the proband (III : 2) showed dMMR with nuclear loss of MLH1 and PMS2 protein, with normal expression of MSH2 and MSH6 protein (Figure 2). Tumour tissue was identified to be MSI-high using microsatellite instability testing (Figure 2). Pathology information was also available from the proband’s uncle (II : 5), whose IHC findings were consistent with the dMMR status of the proband. According to the LS screening guidelines, we also evaluated the *BRAF V600E* mutation status. *BRAF V600E* IHC analysis and genetic testing indicated that this locus had a wild-type sequence. Therefore, we could not exclude this case as hereditary CRC and we recommended the proband for further germline genetic testing of MMR genes.

### Identification of germline MMR genes

**Figure 3.**
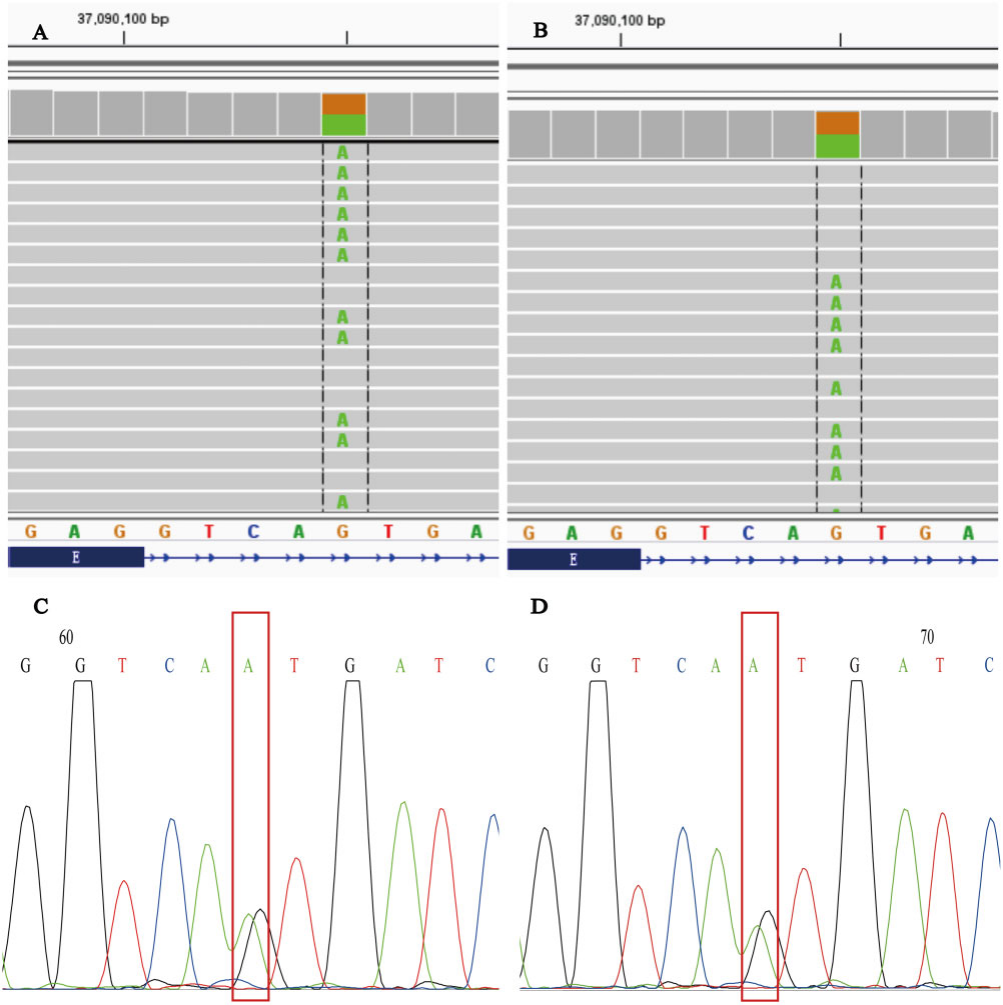
Germline mutation testing by NGS and Sanger sequencing. (A) and (B) NGS results of III : 2 and II : 5. (C) and (D) Sanger sequencing of III : 2 and II : 5.

Germline mutation analysis by NGS in the proband identified a novel variant of uncertain significance in *MLH1* (*MLH1* [NM_000249.3]: c. 1989 + 5 G > A) according to the American College of Medical Genetics and Genomics (ACMG) criteria. It was located in the intron region behind exon 17 of the *MLH1* gene. We performed Sanger sequencing for the family members of the proband. II : 2, II : 5, II : 11, III : 2, II : 3, II : 9, III : 4, III : 5, and III : 6 carried this variant, and II : 7, III : 1, III : 7, III : 9, and III : 10 did not harbour the germline mutation. Among the carriers, II : 2, II : 5, II : 11, and III : 2 also developed CRC. The detailed family pedigree of the proband is shown in Figure 1, and the two patients’ NGS and Sanger sequencing results are shown in Figure 3.

### Assessment of the functional significance of the novel *MLH1* variant

**Figure 4.**
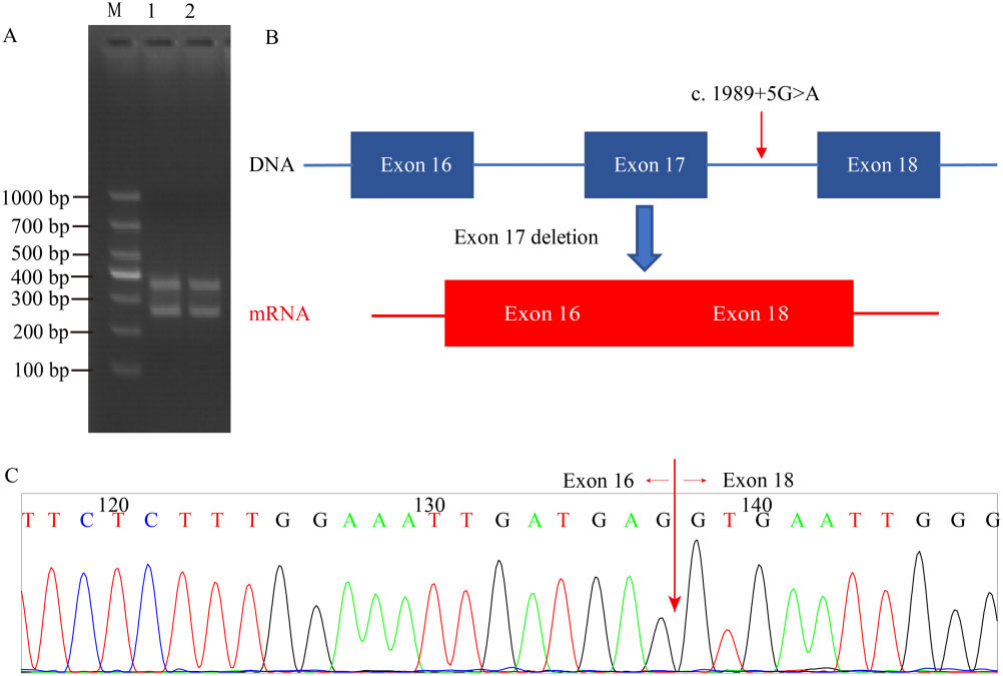
Identification of splicing in *MLH1* c.1989 + 5G>A. (A) Agarose gel electrophoresis. (B) Schematic of splicing. (C) Sanger sequencing of cDNA from the RT-PCR product of total RNA isolated from the peripheral blood of the proband. The arrows indicate mutation at the following nucleotide position of *MLH1* [NM_000249.3]: c. 1989 + 5 G > A.

According to the population-frequency database, *MLH1* c.1989 + 5G>A was absent in the normal control population. Because the variant was near an exon–intron, we evaluated the splicing effects of the variant. *In silico* prediction using Alamut software indicated that the variant resulted in the loss of splice sites in exon 17 and possible skipping of exon 17.cDNA from the RT-PCR product of total RNA isolated from the peripheral blood of the proband was analysed. Agarose gel electrophoresis showed that the PCR products designed to probe *MLH1* exon 17 in the proband had two bands of different sizes, namely 250 and 350 bp. Interestingly, exon 17 was only 93 bp. This was consistent with our hypothesis of *MLH1* exon 17 skipping. Sanger sequencing of the 250-bp product confirmed that there was a fusion transcript of *MLH1* exon 16 and exon 18 without exon 17. A schematic illustration is provided in Figure 4.

## Discussion

Our study found a novel germline pathogenic mutation (*MLH1* c.1989 + 5G>A) related to LS in a patient with a strong family history of cancer. This is an unclassified variant and is designated VUS in the public database. We performed a series of experiments to evaluate the clinical significance as evidence of the ACMG-assessment criteria.

### *MLH1* gene mutation situation

The identification of the pathogenicity of MMR gene mutations is a major problem in genetic testing for LS. Early studies have shown that VUS accounts for a high proportion of MMR gene mutations [11] and that *MLH1* mutations account for the greatest proportion of MMR gene mutations [12]. Recent research has provided more details on this information, showing that *MLH1* accounts for >60% of MMR mutations in LS [13] and that the most common type of mutation in *MLH1* is the change in splice sites [14]. Through NGS and Sanger sequencing, we found that the family members of the proband carried a novel germline mutation (*MLH1* c.1989 + 5G>A). We verified that this intron mutation affected the splicing function of RNA in the transcriptional process, resulting in the splicing skipping of *MLH1* exon 17 and subsequent loss of MLH1 protein expression. This finding enriches the pathogenic mutation spectrum of the *MLH1* gene and provides an interpretation reference for genetic counsellors in LS screening. Thus, the LS-related tumours of patients and their relatives can be accurately managed. Therefore, the incidence and mortality of CRC, which is the major cancer among LS mutation carriers, will decrease.

### Clinical phenotypes and genetic counselling

The average age at diagnosis of LS was 39 years in the first family, which is lower than the median age of 45 years [13]. We found that this mutation-associated CRC occurred at younger and younger ages in families (ranging from parents in their 40s to children in their 20s) and the disease stages were also late. However, we followed up for 60 months and found that these individuals had good prognoses. Our patients with mutations had a high predisposition for CRC, so we should pay much attention to CRC screening. However, according to research [15], Asian ethnicity and *MLH1* mutation carrier status are both high risk factors for Lynch-associated gastric cancer. Therefore, in China, we should pay more attention to the screening of not only Lynch-associated CRC, but also gastric cancer [15]. Among women with *MLH1* mutations, the incidence of endometrial cancer by the age of 75 years is 43% [7]. Hence, the women in this family should also increase screening for endometrial cancer [16]. According to the recommendations of domestic and international researchers, because the tumour-onset age in this family is not <25 years, carriers of the *MLH1* mutation can be screened for gastric cancer by gastroscopy beginning at 30–35 years old and for CRC by colonoscopy every 1–2 years beginning at 20–25 years old [17, 18]. For the screening of gynaecological tumours, women with no fertility requirement can be treated with prophylactic double adnexal hysterectomy. Unoperated carriers without clinical symptoms are advised to undergo an endometrial biopsy every 1–2 years. Regular transvaginal ultrasound examinations for uterine adnexal masses and measurements of serum CA125 can also be considered to reduce the risk of ovarian cancer [18].

### Treatment and prognosis

Studies have confirmed that MMR status can predict biomarkers of immunotherapy [19]. Tumours with MMR deficit may produce a large number of new antigens related to mutations, which can be recognized by the immune system [20]. Hence, immunotherapy has a high response rate among dMMR tumours. In the PD-1 inhibitor study on the efficacy of 12 different and advanced solid tumours with MMR defects [21], the imaging objective remission rate of tumours with MMR deficiency to PD-1 inhibitor treatment was as high as 53% and the imaging complete remission rate was 21%. After neoadjuvant therapy, our patient with *MLH1* mutation benefited greatly from perioperative immunotherapy. Therefore, whether immunotherapy can be considered perioperative adjuvant therapy is an interesting clinical research direction.

### Clinical and molecular diagnostic perspectives

The proband in our study met the Amsterdam criteria. In his parents’ generation, there is a large family pedigree that can provide a family history for him. Nevertheless, there are few family members and a large number of one-child families in China at present. Researchers have shown that according to clinical standards, up to 28% of LS cases might be missed [22]. At the same time, studies show that ∼89% of LS patients meet the revised Bethesda criteria in China, while <20% of Chinese LS patients meet the Amsterdam criteria [23]. Therefore, the characteristics of the family history are less obvious. This makes it more difficult to screen for LS among our Chinese patients. Hence, China should strengthen the molecular detection of LS to improve the identification of this disease. Studies have shown that even if it is non-CRC or non-endometrial cancer, MSI is helpful for the diagnosis of LS patients [24]; however, the reliable way to a definitive diagnosis is to identify the mutated gene, as this will help to guide the treatment of patients and manage cancer risk in carriers. Although at least five genes are associated with LS, ∼50% of suspected LS cases are genetically unknown [23]. Therefore, we recommend that CRC patients with a family history or who fulfil the Amsterdam/Bethesda clinical diagnostic criteria or patients with suspected LS-related tumours undergo screening for MMR gene mutations by genetic testing [13]. NGS has been increasingly used in clinical settings and research to define the diagnosis and explore the significance of other genes in patients with a personal/family history of hereditary CRC. However, there is a lack of research data on Asian populations [25] and we need to continue to explore mutation data in these populations.

## Limitations

Our study has some limitations. In this study, only one pedigree from our single centre was recruited. In the future, other patients with the same mutation need to be included in a large-scale study to further confirm the pathogenicity of the variant.

## Conclusions

In this study, we found a new *MLH1* pathogenic variant (c.1989 + 5G>A). We confirmed that it affected the splicing function of RNA. Therefore, this finding enriches the MMR gene-mutation spectrum of LS and provides a reference for a definite diagnosis, follow-up treatment, and genetic counselling for family members.

## Authors' Contributions

L.L.W., S.M.Z., L.D., and Z.L. designed the research. L.L.W., M.Y., D.Q., and Z.L. performed the experiments. Z.J. and Q.L. provided the study materials and patients. L.L.W., J.N.C., S.W.M., and Z.X.Z. collected the data. X.G. analysed the data. L.L.W., S.M.Z., and L.D. drafted the manuscript. Z.L. and X.S.W. revised the paper. All authors reviewed and approved the final manuscript.

## Funding

This work was supported by grants from the National Key Research and Development Program of China [2018YFC1312100, 2017YFC1311005]. The funders had no role in the study design, data acquisition, analysis, interpretation, writing, or submission of the manuscript.

## References

[1] Ferlay J , Ervik M, Lam F et al *Global Cancer Observatory: Cancer Today*. Lyon: International Agency for Research on Cancer, 2021. https://gco.iarc.fr/today (9 January 2021, date last accessed).

[2] Samadder NJ , Jasperson K, Burt RW. Hereditary and common familial colorectal cancer: evidence for colorectal screening. Dig Dis Sci 2015;60:734–47. 25501924 10.1007/s10620-014-3465-z

[3] Kastrinos F , Stoffel EM. History, genetics, and strategies for cancer prevention in Lynch syndrome. Clin Gastroenterol Hepatol 2014;12:715–27. 23891921 10.1016/j.cgh.2013.06.031 PMC3995833

[4] Møller P , Seppälä T, Bernstein I et al; Mallorca Group (http://mallorca-group.org). Incidence of and survival after subsequent cancers in carriers of pathogenic MMR variants with previous cancer: a report from the prospective Lynch syndrome database. Gut 2017;66:1657–64. 27261338 10.1136/gutjnl-2016-311403 PMC5561364

[5] Ballester V , Cruz-Correa M. How and when to consider genetic testing for colon cancer? Gastroenterology 2018;155:955–9. 30148981 10.1053/j.gastro.2018.08.031

[6] Hampel H , Bennett RL, Buchanan A et al; Guideline Development Group, American College of Medical Genetics and Genomics Professional Practice and Guidelines Committee and National Society of Genetic Counselors Practice Guidelines Committee. A practice guideline from the American College of Medical Genetics and Genomics and the National Society of Genetic Counselors: referral indications for cancer predisposition assessment. Genet Med 2015;17:70–87. 25394175 10.1038/gim.2014.147

[7] Moller P , Seppala TT, Bernstein I et al; Mallorca Group. Cancer risk and survival in path_MMR carriers by gene and gender up to 75 years of age: a report from the prospective Lynch syndrome database. Gut 2018;67:1306–16. 28754778 10.1136/gutjnl-2017-314057 PMC6031262

[8] Chen X , Li X, Liang H et al A new mutL homolog 1 c.1896 + 5G>A germline mutation detected in a Lynch syndrome-associated lung and gastric double primary cancer patient. Mol Genet Genomic Med 2019;7:e787. 31207149 10.1002/mgg3.787 PMC6687634

[9] Karam R , Conner B, LaDuca H et al Assessment of diagnostic outcomes of RNA genetic testing for hereditary cancer. JAMA Netw Open 2019;2:e1913900. 31642931 10.1001/jamanetworkopen.2019.13900 PMC6820040

[10] Richards S , Aziz N, Bale S et al; ACMG Laboratory Quality Assurance Committee. Standards and guidelines for the interpretation of sequence variants: a joint consensus recommendation of the American College of Medical Genetics and Genomics and the Association for Molecular Pathology. Genet Med 2015;17:405–24. 25741868 10.1038/gim.2015.30 PMC4544753

[11] Peltomäki P , Vasen H. Mutations associated with HNPCC predisposition: update of ICG-HNPCC/INSiGHT mutation database. Dis Markers 2004;20:269–76. 15528792 10.1155/2004/305058 PMC3839397

[12] Lindor NM , Guidugli L, Wang X et al A review of a multifactorial probability-based model for classification of BRCA1 and BRCA2 variants of uncertain significance (VUS). Hum Mutat 2012;33:8–21. 21990134 10.1002/humu.21627 PMC3242438

[13] Ow SGW , Tan KT, Yang H et al Next generation sequencing reveals novel mutations in mismatch repair genes and other cancer predisposition genes in Asian patients with suspected Lynch syndrome. Clin Colorectal Cancer 2019;18:e324–34. 31350202 10.1016/j.clcc.2019.05.007

[14] Lagerstedt-Robinson K , Rohlin A, Aravidis C et al Mismatch repair gene mutation spectrum in the Swedish Lynch syndrome population. Oncol Rep 2016;36:2823–35. 27601186 10.3892/or.2016.5060

[15] Wang LL , Liu Z, Wang XS. Progress in Lynch syndrome associated gastric cancer. Chin J Gen Surg 2020;29:1243–50.

[16] Valle L , Vilar E, Tavtigian SV et al Genetic predisposition to colorectal cancer: syndromes, genes, classification of genetic variants and implications for precision medicine. J Pathol 2019;247:574–88. 30584801 10.1002/path.5229 PMC6747691

[17] Balmana J , Balaguer F, Cervantes A et al; ESMO Guidelines Working Group. Familial risk-colorectal cancer: ESMO Clinical Practice Guidelines. Ann Oncol 2013;24 Suppl 6:vi73–80. 23813931 10.1093/annonc/mdt209

[18] Yuan Y. The Chinese expert consensus on clinical diagnosis, treatment and pedigree management of hereditary colorectal cancer. J Pract Oncol 2018;33:3–16. 10.3760/cma.j.issn.0253-3766.2018.01.013 29365422

[19] Park H , Chen B, Ciorba MA. Progress in PD-1-based immunotherapy: new mechanistic insight may provide expanded hope for application to colon and gastrointestinal cancers. Gastroenterology 2017;153:1162–3. 28867277 10.1053/j.gastro.2017.08.050

[20] Zhao P , Li L, Jiang X et al Mismatch repair deficiency/microsatellite instability-high as a predictor for anti-PD-1/PD-L1 immunotherapy efficacy. J Hematol Oncol 2019;12:54. 31151482 10.1186/s13045-019-0738-1 PMC6544911

[21] Le DT , Durham JN, Smith KN et al Mismatch repair deficiency predicts response of solid tumors to PD-1 blockade. Science 2017;357:409–13. 28596308 10.1126/science.aan6733 PMC5576142

[22] Hampel H , Frankel WL, Martin E et al Feasibility of screening for Lynch syndrome among patients with colorectal cancer. J Clin Oncol 2008;26:5783–8. 18809606 10.1200/JCO.2008.17.5950 PMC2645108

[23] Dong L , Jin X, Wang W et al Distinct clinical phenotype and genetic testing strategy for Lynch syndrome in China based on a large colorectal cancer cohort. Int J Cancer 2020;146:3077–86. 32030746 10.1002/ijc.32914

[24] Latham A , Srinivasan P, Kemel Y et al Microsatellite instability is associated with the presence of Lynch syndrome pan-cancer. J Clin Oncol 2019;37:286–95. 30376427 10.1200/JCO.18.00283 PMC6553803

[25] Cragun D , Radford C, Dolinsky JS et al Panel-based testing for inherited colorectal cancer: a descriptive study of clinical testing performed by a US laboratory. Clin Genet 2014;86:510–20. 24506336 10.1111/cge.12359 PMC4127163

